# Towards anti-racist futures: a scoping review exploring educational interventions that address systemic racism in post graduate medical education

**DOI:** 10.1007/s10459-024-10343-1

**Published:** 2024-06-14

**Authors:** Baijayanta Mukhopadhyay, Vivetha Thambinathan, Elizabeth Anne Kinsella

**Affiliations:** 1 Office of Social Accountability and Community Engagement, Faculty of Medicine and Health Sciences, Institute of Health Sciences Education, McGill University, Montreal, QC Canada; 2 Institute of Health Sciences Education, Faculty of Medicine and Health Sciences, McGill University, Montreal, QC Canada; 3 Department of Equity, Ethics and Policy, Faculty of Medicine and Health Sciences, Institute of Health Sciences Education, McGill University, Montreal, QC Canada

**Keywords:** (4–6) Anti-racism, Structural racism, Systemic racism, Medical education, Medical intervention, Residency education

## Abstract

Since 2020, brought to the forefront by movements such as Black Lives Matter and Idle No More, it has been widely acknowledged that systemic racism contributes to racially differentiated health outcomes. Health professional educators have been called to address such disparities within healthcare, policy, and practice. To tackle structural racism within healthcare, one avenue that has emerged is the creation of medical education interventions within postgraduate residency medical programming. The objective of this scoping review is to examine the current literature on anti-racist educational interventions, that integrate a systemic or structural view of racism, within postgraduate medical education. Through the identification and analysis of 23 papers, this review identified three major components of interest across medical interventions, including (a) conceptualization, (b) pedagogical issues, and (c) outcomes & evaluation. There were overlapping points of discussion and analysis within each of these components. Conceptualization addressed how researchers conceptualized racism in different ways, the range of curricular content educators chose to challenge racism, and the absence of community’s role in curricular development. Pedagogical issues addressed knowledge vs. skills-based teaching, and tensions between one-time workshops and integrative curriculum. Outcomes and evaluation highlighted self-reported Likert scales as dominant types of evaluation, self-evaluation in educational interventions, and misalignments between intervention outcomes and learning objectives. The findings are unique in their in-depth exploration of anti-racist medical interventions within postgraduate medical education programming, specifically in relation to efforts to address systemic and structural racism. The findings contribute a meaningful review of the current state of the field of medical education and generate new conversations about future possibilities for a broader anti-racist health professions curriculum.

**Supplementary Information:**

The online version contains supplementary material available at 10.1007/s10459-024-10343-1.

## Introduction

Scholars, activists, and clinicians have scrutinized racially differentiated health outcomes for many years (Otu et al., 2020; Mugglin & Ruedin 2022; Ramraj et al., 2016; Bleich et al., 2012). 2020 saw intensified focus on these realities, when the deaths of George Floyd in the United States, Joyce Echaquan in Canada, and the inequities evident in the Covid-19 pandemic spawned movements demanding that institutions across society, including healthcare, respond to such disparities (Chinekezi et al., 2023; Osei-Tutu et al., 2023; Rahman-Shepherd et al., 2023; Razack & Naidu, 2022). An important root cause of health inequity is the unequal systematic allocation of resources, funding, and power, which manifest in unequal social determinants of health across society (Baciu et al., 2017). Health professional educators have been called upon to prepare future health workers so that they are equipped to meet society’s evolving expectations to address these inequities that are now understood not to be inevitable, but produced by practices, procedures, and policies that structure our communities and societies (Hardeman et al., 2016). These patterns result in tangible inequities in health outcomes, resulting in disproportionate mortality and morbidity in some groups over others. In underlining the structural nature of inequities, Ruth Wilson Gilmore’s definition of racism can be understood to implicate healthcare directly: “Racism, specifically, is the state-sanctioned or extralegal production and exploitation of group-differentiated vulnerability to premature death” (2007, p.50). As educators and scholars attempting to create and implement educational programming in postgraduate medicine that addresses structural racism, we are inspired by Wilson Gilmore’s formulation to ask: *How do we teach to produce clinicians with skills to undo the production and exploitation of group-differentiated mortality?* The aim of this scoping review is to document the current state of knowledge about interventions which aim to equip medical residents with capabilities to address structures of racism that influence health outcomes. For the purposes of this paper, we use the terms “systemic racism” and “structural racism” interchangeably, as they are often used in this way in the literature we examined. It should be noted however that some scholars depict systemic racism as a broader concept encompassing whole systems (i.e. political, legal, economic, health care, school, and criminal justice), and structural racism as focused to a greater extent on the structures (laws, policies, institutional practices, and entrenched norms) that uphold these systems (Braveman et al., 2022).

We focus on postgraduate medical education (PGME/residency) for several reasons. During residency, clinical training is sustained and intense, and learners develop knowledge that becomes foundational to clinical life as they make key career choices (Yang et al., 2022). This critical pedagogical moment (PGME) offers a potent space for approaches to education that promote antiracist practice to be introduced. It is well known that the individual clinical encounter has limited influence on broader health outcomes, which are also determined by structural factors outside of the clinical setting, such as access to housing, nutrition, social networks, and state support (Hood et al., 2016). Entrenched racism shapes these factors and needs to be considered in efforts toward anti-racist practice (Thambinathan & Kinsella, 2023). As such, a focus on interpersonal interactions in the clinical setting, while important, is not sufficient in improving anti-racist practice and transforming outcomes in practice.

Previous reviews have tackled questions about how to develop skills to address racism in medical education; with racism being defined through various analytic lenses such as equity, cultural competency, or cultural safety. We have identified an absence in literature that specifically consider racism through a structural or systemic lens, or that focus particularly on interventions in postgraduate medical education.

Some reasearchers have studied graduate medical education with a focus specifically on diversity, equity, and inclusion. Chung et al. (2023) look broadly at diversity, equity, and inclusion related programming across residency curricula. Hunter and Thomson (2019) look broadly at the incorporation of education about the social determinants within graduate medical education, and Gard et al. (2019) explore the same within primary care residency programs. A limitation however is that they do not explore the definitional boundaries of their terms in depth or delineate what skills are being targeted to address racism at which levels; thus, none specifically address structural racism and associated health outcomes.

Several recent reviews of health education to address racism are situated in disciplines outside of medicine. For instance, Collins et al. (2023) looked at the incorporation of critical race theory within public health education, and Narasimhan et al. (2021) studied efforts toward decolonization within public health related to Indigenous health behaviour and education. Perkins et al. (2023) and Al-Shakarshi et al. (2019) reviewed education around concepts of colonialism and equity within global health education respectively. Various concepts around anti-racism, equity, and cultural safety or competency have been the focus in health professional or interprofessional education at various levels in different geographies, some specific to Australia or Canada, others more broadly regional or global (Brottman et al., 2020; Brumpton et al., 2022; Dowell et al., 2022; Guerra & Kurtz, 2017; Jongen et al., 2018; MacLean et al., 2023). Anti-racist education is discussed in several reviews in disciplines such as nursing (Červený et al., 2022; Gradellini et al., 2021; Oikarainen et al., 2019), midwifery (Capper et al., 2022), or rehabilitation professionals (Grandpierre et al., 2018). Biles et al. (2022) discuss the literature around Indigenous community involvement in health research in Australia. Other articles explore racism in undergraduate medical education specifically and adopt a wide range of analytic frameworks such as social determinants of health, or cultural competency (Deliz et al., 2020; Doobay-Persaud et al., 2019; Nour et al., 2023; Rashid et al., 2020).

Thus, given the rise in consciousness about racism in healthcare and the gap in literature focused on exploring and documenting systemic and structural anti-racism educational interventions in postgraduate medical programs, we position this scoping review to examine the current state of knowledge about educational interventions in postgraduate medical programs which address systemic or structural racism in medicine. As a research team, we made the assumption that educational interventions that address systemic or structural racism would inherently fall under the category of ‘antiracist’ approaches, even if this was unintentional or implicit. This may reflect our professional and personal worldviews that tackling racism requires embedded anti-racist pedagogies and practices best described by Dr. Angela Davis’ powerful quote “In a racist society it is not enough to be non-racist, we must be anti-racist” (Davis, 2012). As authors of this paper, we position ourselves as medical education researchers and practitioners committed to the professional responsibility of anti-racism work within the academy and are dedicated to shifting our power and privilege in ways that stand against institutional racism. From this basis, we attempt to portray anti-racism educational interventions through the lens of who is designing, what makes up the content, and how is it taught, and propose ways forward to focus more strategically on the skills and knowledge physicians need to address health injustices in the communities they provide care for.

## Methodology

In this scoping review, we examine the current literature on anti-racist educational interventions, that integrate a systemic or structural view of racism, within postgraduate medical education. The aim was to identify, examine, and summarize the peer-reviewed and grey literature about educational interventions in this area. The scoping review protocol followed the methodological framework outlined by Arksey & O’Malley (2005) detailed below.

### Stage 1: identifying the research question

The following research question guided the review:

What is the current state of knowledge about educational interventions in postgraduate medical programs that address systemic or structural racism in medicine?

### Stage 2: identifying relevant documents

Eligibility criteria were used to identify relevant literature. The inclusion criteria were as follows: studies examining anti-racism interventions instituted by medical faculties in residency education programs; study designs that included an educational intervention addressing systemic and structural racism; peer-reviewed research studies (formal reviews (ie. scoping reviews, systematic reviews, etc.), experimental, quasi-experimental, observational, qualitative); grey literature (theses, editorials, commentaries, narrative reviews); publications from 2010 or later; and studies in English. All peer-reviewed research studies (experimental, quasi-experimental, observational, qualitative) were included; we did not restrict studies based on study design.

The exclusion criteria consist of: educational interventions not conducted through a medical institution or faculty; educational interventions not integrated into a residency education program; study designs that did not include an anti-racism educational intervention; educational interventions that focused solely on interpersonal racism, without mention of structural or systemic inequities; studies conducted outside of Australia, Canada, Europe, New Zealand, and the United States; publication prior to 2010; and languages other than English. Other non-Western areas of the world were excluded given the particular histories they have faced where white supremacy and racism may express themselves differently in clinical practice, which would ultimately impact the scope and focus of this review. We limited publication date range from 2010 until present-day to ensure the medical education interventions were not outdated.

#### Search strategy

The search strategy was iteratively developed by the research team in consultation with a health sciences education librarian. This strategy was initially built in Medline and then adapted as required for the other databases. Our search string includes: exp racism/OR Racism or Implicit bias or Race or Cultural Competenc* or cultural safety or cultural humility or cultural sensitiv* or Implicit racial attitudes or Racial bias or White supremacy).mp., AND education, medical/OR (Medical education or Medical school* or Medical facult*).mp. AND (Toolkit or Program or Training or Workshop or Curricul* or Course).mp. We searched the following databases: medline-Ovid, EMBASE, PsycINFO, CINAHL, Theses/Dissertations (ProQuest), ERIC (ProQuest), and Web of Science Core Collection. This range of search terms and databases informed a comprehensive search.

**Fig. 1:**
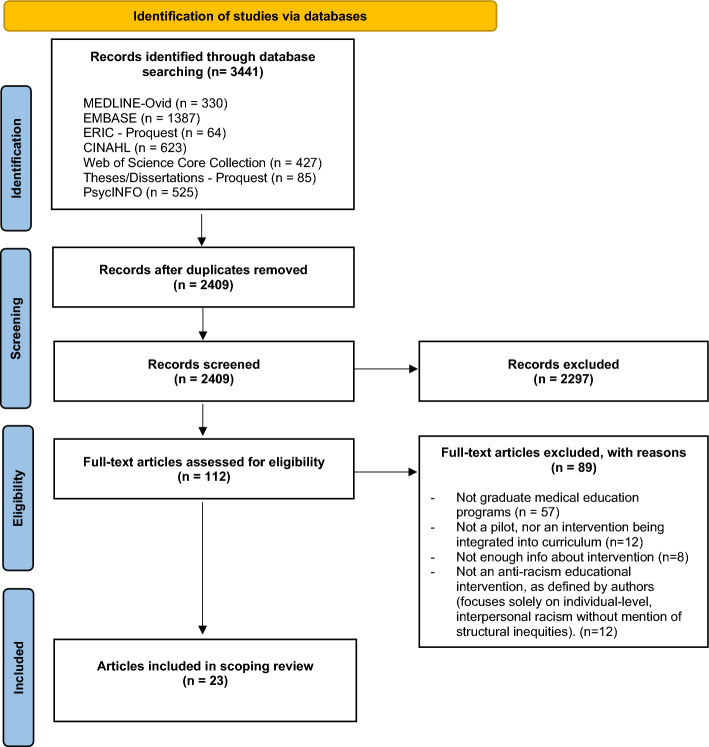
PRISM flow diagram. (Tricco et al., 2018)

The search strategy included both subject headings and keywords related to racism, curriculum, and medical education (see Additional file 1). In addition, a grey (unpublished) literature search was conducted using ProQuest Dissertations and Theses Global. The results were exported into the citation manager EndNote to retrieve bibliographic details and remove duplications. A total of 3441 peer reviewed and grey literature titles were retrieved from the search, 1032 duplicates were removed resulting in 2409 titles (see Fig. 1 for PRISMA diagram).

### Stage 3: study selection

The study selection process consisted of two rounds of screening: title and abstract; full-text review. Rayyan online software was used as a screening and data extraction tool during this process. To start the screening process, BM and VT engaged in a sensitizing exercise by reviewing 10 articles together and discussing what would be included and excluded, to familiarize each other with applying the screening criteria. Then in the title/abstract stage, BM and VT independently reviewed the articles and determined eligibility using the eligibility criteria. When consensus was not achieved, the two reviewers consulted with EAK to resolve disagreements. Through screening of 2409 documents, 2297 were removed because they did not meet the inclusion criteria resulting in 112 full text documents. The full-text documents were reviewed by VT, who applied the same criteria and set aside and reviewed any unclear documents with BM and EAK; 89 were excluded due to ineligibility, and 23 articles were included in the review. The PRISMA flow diagram (see Fig. 1) summarizes the search strategy results and eligibility screening process.

### Stage 4: data items and data collection process

A data charting form was developed to extract data relevant to our research questions into Excel. The data charting form was piloted and reviewed by the research team to ensure attention to important data points. The following information was extracted and recorded in the form.

a. Descriptive information: authors, title of article, title of publication, year of publication, country of origin, publication type, study aims/purpose/goals, research study design, study population and setting, description of educational intervention, outcome measures, key findings, and study limitations.

b. Anti-racism conceptualization: presence of ‘anti-racism’ term in document, anti-racism definitions and conceptualizations, and key takeaways/thought-provoking notes.

### Stage 5: synthesizing and reporting the results

A descriptive analysis and thematic analyses were undertaken. For the descriptive analysis, we collated the data according to various descriptive categories. The thematic analysis took place amongst all three team members. Initially, each researcher independently created a mind map of themes and subthemes arising from analysis of the data. This process allowed for the team to brainstorm key ideas related to the themes and draw connections between them (Tattersall et al., 2011). Research team meetings occurred throughout the data extraction process, with new themes and categories of interest emerging through an iterative process of thematic analysis and dialogue. Themes and subthemes were identified, integrated, and a thematic map of findings was collaboratively and iteratively generated. Any disagreements in interpretations were negotiated through dialogue between the research team.

## Results

### Descriptive analysis

We identified 23 papers that addressed anti-racism within postgraduate residency programs using faculty-led educational interventions (Additional file 1). The papers were published between 2010 and 2022 with the majority published in the last four years: 2022 (7 papers), 2021 (4), 2020(1), 2019 (5), 2016 (3), 2015 (1), and 2010 (2). Twenty-two papers were from the United States, and one from Switzerland.

Twenty-one of the articles discussed specific educational interventions aimed at residents. The intervention studies focused on residents in family/community or preventative medicine (5), psychiatry (3), emergency medicine (2), paediatrics (2), internal medicine (1), and neurology (1); as well as combined residency intervention programs in internal medicine & emergency medicine (1), internal medicine & pediatrics (1), family medicine, emergency medicine, & surgery (1). Four programmes focused on all residents across specialities in a hospital. One included practicum rotations in community settings. One additional paper was a scoping review on cultural competency curricula in PGME with a focus on anti-racism interventions. Another was a theoretical paper that proposed components of an educational curricula on cultural competency—with a focus on anti-racism—in residency education in psychiatry.

The majority of types of educational interventions included a combination of didactic sessions and interactive workshops. We also found examples of simulation encounters, a museum visit, community visits, role plays, and a journal club. The articles described a mix of interventions led by faculty (14), or residents (4), or by both groups in collaboration through a task force or working group (3). The length of time of the interventions varied from one-time sessions ranging from one to three hours in duration (8) to a short series of programming consisting of several weeks, while others were structured over a longer period of up to 18 months.

## Conceptualizations

### Researchers conceptualized racism in different ways

Of the 23 studies, there were clear clusters of how racism was conceptualized. Nine papers explicitly discussed the structural nature of racism, some explicitly making the distinction between interpersonal racism, pointing to how social and organizational structures are implicated in health outcomes such as economic, educational, and judicial systems and how they distribute resources (Drum et al., 2021; Garvey et al., 2022; Jindal et al., 2022; Johnson et al., 2022; Karvonen et al., 2022; Mendizabal et al., 2021; Neff et al., 2017; Sherman et al., 2019; Smith et al., 2021). Structure was highlighted through issues like policies and practices of economic, educational, and judicial systems, and their impact on health (Neff et al., 2017), policing as contributors to racialized inequities in health (Smith et al., 2021), and the importance of redistribution of resources in achieving racial equity (Karvonen et al., 2022). For Johnson et al (2022), systemic racism “creates a structural framework in a society utilizing a system of racial hierarchy…that determines access to the social and material necessities of life” (p. 141), and is distinct from interpersonal processes such as implicit bias. Jindal et al. (2022) also underline the distinction between structural and interpersonal racism. Garvey et al. (2022) argue that race is constructed to further exploitative hierarchies and is not a phenomenon that inherently exists in the world. For Mendizabal et al. (2021), the systemic distribution of disparities in health are clearly linked to racism. And Sherman et al. (2019) specifically locate implicit bias as part of the larger system of oppression within which healthcare operates.

Another set of papers did not explicitly discuss racism as embedded within structures and processes within society at large but acknowledge the factors and determinants that contribute to health. These papers often use the language of health disparities and social determinants; here, racism was coded as determinants of health leading to racially inequitable outcomes. As one example, a paper by Simpson et al (2022), focuses on racism’s impact on health through “psychophysiological pathways” suggesting that responses to stressors by individual biology cause health harm. This contrasts a focus on the maldistribution of resources and power in a racialized hierarchy as the cause of sickness. A paper by Willen et al. (2010) also falls into this category, where even though structures are not identified, racism’s contributions to health disparities are. Mian et al. (2010) discusses the historical racist underpinnings of transcultural psychiatry and the contribution of social and economic determinants of health disparities, yet do not explicitly discuss racism as a structure shaping people’s health. Chary et al (2021) focus on racism at the level of the institution, but do not go further to explore policies and patterns outside of hospitals. A scoping review by Atkinson et al. (2022) also includes language around the social determinants of health in relation to race and ethnicity however does not explicitly consider structural racism. In contrast, a paper by Dennis et al. (2019) focused solely on racism within interpersonal relationships.

Some papers did not acknowledge racism in their definitions, focusing instead on social, cultural or non-medical influences on health disparities (Diaz et al., 2016; Kokas et al., 2019; Dennis et al., 2019; Zeidan et al., 2020). While these papers did not acknowledge racism in their definitions, they did attend to anti-racist approaches in the educational interventions or practices described. To us, this language of “disparities” seemed to code for structural racism, as inequities that extend beyond the interpersonal.

In two recent papers, racism was conceptualized as intersectional, as the interventions sought to develop skills to address equity across many different factors, including racism, which they do not specifically explore, but mention as a determinant of health disparities (Emery et al., 2022; Martinez et al., 2021). Another paper did not specifically describe racism but discussed it in working across healing traditions and communities that survived colonialism (Kesler et al., 2015). The paper by Paroz et al. (2016) drew an interesting link between immigration and growing disparities in class structures and the impact on health disparities.

### Educators chose a range of curricular content to challenge racism

Distinct from how they describe racism, curricular choices in what is taught reveal frequent misalignment between how educators define the issues at stake, and the educational content provided to trainees. Only two studies linked the structural problem of racism to the education interventions offered. For instance, the structural competency educational intervention (Neff et al., 2017) clearly focuses on the systemic nature of racism and its impact on health; and another educational intervention focuses on the structural nature of racism by asking residents to operationalise anti-racist practice by applying a specific tool to critically appraise medical evidence (Garvey et al., 2022).

Other interventions (Drum et al., 2021; Kokas et al., 2019; Paroz et al., 2016; Zeidan et al., 2020) that explicitly frame racism as a more systemic or structural issue, include curricula that also focus on interpersonal dimensions of racism, such as microaggressions and implicit bias. Some examples include: teaching about white identity (Drum et al., 2021; Sherman et al., 2019) and a journal club that integrates the discussion of many structural issues such as Covid-19’s impact on minoritized populations, wait-time disparities, access to care, health insurance status with more interpersonal ones like implicit bias (Smith et al., 2021).

The study by Willen et al. (2010) does not explicitly mention racism as a structural force, but their curriculum addresses it by covering social and economic factors shaping the mental health clinical encounter, and by taking a critical look at how the field of psychiatry contributes to these dynamics. In contrast, Simpson et al. (2022) address race as a social construct in the content of their workshop, including the distinct levels at which it acts, but the tools used appeared to only interrupt racism on an interpersonal level.

The majority of remaining interventions address a mix of both structural and interpersonal expressions of racism in the content of what is taught (Diaz et al., 2016; Dennis et al., 2019; Mendibazal et al., 2021; Chary et al., 2021; Johnson et al., 2022; Jindal et al., 2022;. Two interventions however are of note. The intervention detailed by Johnson et al. (2022) was unique in that their curriculum was part of a concerted attempt to change their residency programme’s climate around issues of equity, and some analysis of structural racism within the curriculum was offered. The paper by Kesler et al. (2015) is intriguing in that the curriculum is not so much focused on racism, but the educational pedagogy decentres the EuroAmerican model, and actively embraces a relationship with another healing tradition (Curanderismo)—such that it seems to be doing anti-racist work, rather than teaching it.

The interventions presented by Emery et al. (2022) and Martinez et al. (2021) do not focus specifically on anti-racism but rather teach skills that aim to incorporate anti-racism into trainees’ practice. Similarly, Mian et al. (2010) do not offer curricular tools that address a structural analysis of racism and its impact on mental health, although they implicitly refer to such an understanding in their background.

### Absence of community’s role in curricular development

Most papers showed no evidence of community partner or patient group participation within the curriculum development or delivery. Overall, the paper by Kesler et al. (2015) appears to have the most developed role for community members in the development and delivery of their programme, with teachers with lived experience, and experiences embedded with the community health organisations as examples. Neff et al. (2017) note the contribution of community health activists to the development of the residency training, and Paroz et al. (2016) describe how simulated patients and cultural competency trainers were involved in the development of an education module.

Some other studies show some consideration of community context and impact, but with no explicit mention of direction by community members (Smith et al., 2021; Diaz et al., 2016; Mendibazal et al., 2021; Kokas et al., 2019; Emery et al., 2022). In the paper by Karvonen et al. (2022), while there was no apparent community input into the curriculum design or evaluation itself, a larger initiative to transform the residency experience alluded to partnering with community organisations in the hospital’s neighbourhood.

## Pedagogical issues

### Knowledge versus skills-based teaching

Though the overall educational goal of all interventions was to change clinical practice, one of the key distinctions in the pedagogy of curricular interventions was between knowledge based interventions and skills based interventions. The studies showed a range of approaches along this continuum, with a predominance of studies in the first category suggesting a potential misalignment between goals and pedagogical approaches.

Some studies focused solely on reinforcing knowledge and failed to address skills (Smith et al., 2021; Jindal et al., 2022; Chary et al., 2021; Zeidan et al., 2020). Others, like Simpson et al. (2022) held discussion sessions on how to address racism in practice but did not appear to work on skill-building. The novel pedagogical intervention described by Dennis et al. (2019) focused on knowledge building, with residents’ reflective evaluations stating that it fostered critical self-awareness and would lead to advocacy and education. However, they do not mention changes in *clinical practice.*

Other interventions spanned knowledge and skills, although few directly addressed ways to change clinical practice. For example, both Drum et al. (2021) and Karvonen et al. (2022) describe interventions that included a component on skills for responding to microaggressions, that focuses on the interpersonal and could also be interpreted as focusing on collegial relationships rather than therapeutic relationships. Kokas et al. (2019), Mendizabal et al. (2021), and Diaz et al. (2016) offered the opportunity to improve interviewing strategies in their interventions.

Martinez et al. (2021) state that their intervention intended to improve skills in allyship with underrepresented communities, although the pedagogy described includes an hour of case discussion that does appear to explicitly model or practice skills. The interventions by Johnson et al. (2022) and Sherman et al. (2019) incorporated some skill-building, such as in community health assessment and in active listening, however it was unclear whether these would translate directly into clinical practice. Willen et al. (2010) acknowledge that their emphasis on skills-building was less than other more knowledge-based goals, however improving clinical diagnostic skills in how to recognize psychiatric distress across a wide range of cultural expressions, was a key focus.

Some articles describe very skills-oriented curricular interventions, ranging from critical appraisal to leadership and communication skills (Emery et al., 2022; Garvey et al., 2022; Kesler et al., 2015; Neff et al., 2017; Paroz et al., 2016).

In the remaining studies, Hammond and James (2020) describe an experiential educational intervention that develops the ability to discuss racism through the experience itself. Mian et al. (2010) discuss the importance of developing skills to build responsive care across cultures, beyond a conceptual understanding alone. The review by Atkinson et al. (2022) highlights two pedagogical issues related to knowledge-based and skills-based training: namely, that the limited long-term retention from many interventions suggests that clinical practices may not change in meaningful ways, and that people may actually feel overwhelmed by knowledge about the structural realities if they do not have skills to address them.

### Silos & systems: navigating between one-time workshops and integrative curriculum

Educational interventions varied in the length of time, ranging between one-time workshops, a short series of programming lasting several weeks, and longitudinal curriculum content structured for a year or longer. Eight out of 19 interventions were one-time workshops, where content delivery occurred during a single planned session, separate from the medical curriculum (Chary et al., 2021; Emery et al., 2022; Garvey et al., 2022; Kokas et al., 2019; Martinez et al., 2021; Neff et al., 2017; Simpson et al., 2022; Zeidan et al., 2020). These consisted of a combination of didactic lecture, video components, case studies, and reflective discussion in small groups. One workshop was notably different in its pedagogy with a focus on interactive exercises and reflective discussion following a pre-briefing, rather than a didactic lecture (Zeidan et al., 2020). This intervention was also unique in its deliberate move to a location ‘outside’ of the clinical environment; the authors explain “we postulated that decoupling the training from the hospital and direct clinical experiences and having the discussions in a low-stakes environment, such as a museum, could be an effective way to introduce the concept” (Zeidan et al., 2020, p.1).

Two interventions fall somewhere between a one-time workshops and integrative curriculum, (Drum et al., 2021; Jindal et al., 2022). One intervention used reflective discussion and perspective-taking exercises in advance of three 1 h lectures, with a focus on addressing racism in knowledge, motivation, skills, and behaviours (Jindal et al., 2022). Another month-long academic elective seemed to have an integrative approach, yet after close reading, the anti-racism component made up only two lectures (Drum et al., 2021). Limited resources may contribute to decisions to offer one-time workshops but these may still be beneficial for residents with little background on such topics. Some studies considered *when* the workshop would be most effective in the curriculum. Emery et al. (2022) targeted educational intervention towards incoming residents prior to their intern orientation. Many papers described their workshop as occurring during a certain didactic series or residency conference, (Chary et al., 2021; Garvey et al., 2022; Martinez et al., 2021). The remaining articles did not specify when the intervention occurred within the curriculum.

In contrast to one-time workshops, the integrative curricular interventions had stronger content and pedagogical design elements and dedicated more time toward the application of the skills and tools being taught. For example, in the Health Equity journal series by Smith et al. (2021), the educational content was identified and mapped to the ACGME program, core competency milestone 2.0 requirements, the Joint Commission’s institutional recommendations of culturally competent care, and clinical learning environment mandates. When possible, journal club content aligned with national observances. Furthermore, residents volunteered to moderate sessions and were mentored by the taskforce. A key pedagogical design was how the sessions ended with the group brainstorming projects that could be incorporated into their residency and would address disparities identified during the session. Similarly, Diaz et al. (2016) implemented an integrated curriculum that adopted innovative pedagogical strategies, such as the ‘resident as teacher’ construct, creating video vignettes, role-playing, and experiential learning. Karvonen et al. (2022) used standardized simulations on interrupting microaggressions, as well as racial affinity groups, which are separate spaces for people who share a racial identity to gather and share experiences with each other. Likewise, Johnson et al. (2022) included a narrative medicine component to strengthen resident empathy and connection to patients, as well as an advocacy component through which a local journal club and community assessment project emerged. In particular, the intervention described by Kesler et al. (2015) is the most comprehensive, and includes: an interactive course about traditional medicine, progressive practicum rotations that allowed residents to be the educators, a practicum rotation at a clinically based integrative medicine centre, and an applicable online curriculum. A variety of different instructional strategies were used for each curriculum component, such as resident-directed projects, preceptor teaching and modeling, case studies, etc. These were found to be memorable by residents, as they were long-term and sustainable education interventions.

## Outcomes & evaluation

### Types of evaluation: the predominance of self-reported likert scales

Out of the 19 graduate-level medical educational interventions in our review, only two were not evaluated (Smith et al., 2021; Hammond et al., 2020). Thirteen of 19 interventions were evaluated quantitatively, using Likert scale surveys (1, 3–6, 9–11, 13, 14, 16, 19, 21). Of these, 9 also included a qualitative evaluation component, with the tools including: qualitative comments, open-ended questions, free-text comments, and focus groups (1, 3, 6, 10, 11, 13, 14, 19, 21). Four of 19 interventions adopted a solely qualitative approach to evaluation, with data collection methods such as written reflective feedback, semi-structured interviews, and focus groups (Dennis et al., 2019; Jindal et al., 2022; Neff et al., 2017; Willen et al., 2010). Low response rates and small sample sizes were key limitations described in many of the evaluations. Only one study reported on the representation of evaluation respondents. Like other evaluation respondent demographics, Mendizabal et al. (2021) found that respondents were mostly white, had at least one parent with a professional or doctorate degree, and had a childhood household income > $100,000. This profile is especially concerning in anti-racism interventions, but may also reflect the demographic make-up of residents in this study.

### Self-evaluation in educational interventions

Fifteen out of 17 evaluations of educational interventions were completed by participants themselves, raising questions about whether self-evaluation in this area of medical education is sufficient to demonstrate that the intervention works. Jindal et al. (2022) identified self-reported findings as a limitation, citing that it “may not translate to skill development in the real-world context” (p.340). Two interventions adopted innovative approaches to evaluation. First, a preventive residency that integrated traditional healing in its curriculum used a multi-pronged method to evaluation (Kesler et al., 2015). This was completed through measuring resident performance (evaluations, examinations), program quality (resident evaluations of activities), graduate performance (quality improvement survey), with resident knowledge and attitude being evaluated by individual mentors. What stands out is the evaluation of resident performance, including knowledge and attitudes, by another individual. Mimicking the way other aspects of medical education is graded, this type of evaluation may be important for generating critical feedback to improve performance. A second approach used an external evaluator with expertise in the evaluation of social change-based programming to evaluate transformative implicit bias training (Sherman et al. (2019). “Recognizing that measuring implicit bias and the change in bias quantitatively is problematic” Sherman et al.’s (2019) approach to evaluation used focus groups (p.678). The independent evaluator asked questions of the groups related to participants’ experiences with the educational intervention, impacts of the intervention on individual roles and on the broader residency program, and areas of growth related to implicit bias. Though this intervention still used a form of individual self-reflection, the phenomenological qualitative approach to evaluation evoked deeper, critical thoughts and experiences than traditional Likert scale survey questions.

### Intervention outcomes and learning objective (mis) alignment

Many of the interventions evaluated outcomes that were not aligned with their outlined learning objectives. Though many interventions aimed for residents to learn, develop, and utilize skills around addressing systemic and structural racism in practice, the outcomes evaluated revolved primarily around knowledge, attitudes, and perceptions of the interventions. In a paper by Emery et al. (2022) discussing a cross-institutional community organizing workshop for residents, the authors noted, “although we assessed participant’s reactions, we did not assess any impacts on their skills or knowledge and were therefore unable to evaluate several of our educational objectives” (p.5). Quantitative survey outcome measures of interventions primarily evaluated: value added to education, level of awareness, self-efficacy in managing care, comfort level, satisfaction with workshop, etc. (Drum et al., 2021; Diaz et al., 2016; Johnson et al., 2022; Garvey et al., 2022). Despite these measures’ ability to provide insight into the levels of residents’ knowledge, attitude, and perception, the translation from knowledge to practice was not captured. This suggests a disconnect where the learning objective is to impact residents' clinical practice, but the outcome being measured is the impact as perceived by the resident. Between the stages of curricular learning objectives and evaluation of intervention, the studies’ outcomes are often unclear. Typically, these studies do not end up measuring the impact of the interventions on clinical practice, or on patients and communities. Ongoing conversations about objectives and outcomes throughout the design and evaluation phases of education interventions can be helpful.

In contrast, some interventions show that the designers have thought critically about impact. Specifically, qualitative approaches to evaluation provide richer insights into measuring learners’ translation from knowledge to practice, as well as evaluating the change in their clinical practice, based on what they gained from the educational interventions. The open-ended nature of qualitative research possesses the potential of filling in these missing pieces, thereby indicating a closer alignment between interventions’ outcomes and their outlined learning objectives. The study by Jindal et al. (2022) describes that their “methodology did not allow for evaluation of observed behaviours or health outcomes, which is the gold standard” (p. 340). However, they did elicit participants' self-reported action planning, which they state occurs prior to behavior change. Examples include participants discussing perceived impact as it relates to perceived development of skills to mitigate bias, turning insight into strategies to combat racism and improve patient care, and contextual factors that impede or promote behaviour change (Jindal et al., 2022). In another study evaluating learner reactions to activities exploring racism as a social determinant of health, a more direct approach was used to measure outcomes. Through written feedback, participants answered the question: “How will you incorporate what you learned during this monthly meeting into your project, studies, and other aspects of your life?” Rather than measuring the intervention’s impact on their comfort, self-efficacy, or knowledge, these studies address the aforementioned gold standard: the evaluation of observed clinical practice or patient/community health outcomes.

In one structural competency training for medical residents by Neff et al. (2017), post-session surveys and focus groups were used to gather feedback on the training content, experience, effectiveness, and impacts on clinical practice post-training. However, the authors disclose: “our evaluation addresses neither the longevity of this impact nor the potential effects of incorporating structural competency curricula longitudinally” (Neff et al., 2017, p.432). On this issue of long-term retention, three studies from a scoping review by Atkinson et al. (2022) found that residents’ awareness of their own privilege significantly declined over time, while other multicultural knowledge, skills, and attitudes also decreased but did not achieve significance.

## Discussion

### Definitions/conceptualizations

A key issue identified in the review is how definitions of racism and its structures need further clarification within anti-racist educational interventions in PGME. Although our review focused on approaches that address structural racism, the ways in which these ideas were conceptualized varied considerably. While this breadth may be a response to the many-headed hydra nature of systemic racism, a notable finding in the results is the lack of consistency between what is defined as the impact of structural racism in medicine, and what trainees are trained to act upon. Developing pedagogical clarity will enhance future educational initiatives by focusing which competencies need to be developed to address racially-differentiated health outcomes. As discussed in the introduction, understandings of racism that focus only on interpersonal dimensions are limited because they overestimate the impact of a health professional’s behaviour on long-term health outcomes. Interventions that remain congruent in definition and in practice when they articulate the ways in which racism structures access to material and social necessities, as described by Johnson et al (2022), provide conceptual scaffolding that allows transformative practice to take root. Health professionals need to understand the tangible effects of white supremacy on their patients’ experiences of illness and treatment. Conceptualizations that obscure these direct effects by couching them in diffuse “determinants” make it less clear to trainees where they are situated within the larger constellation of systemic factors that build structural racism, and what specific targets in active policy choices that maintain these systems they might be able to act upon.

An integrated framework for teaching anti-racism at different levels, that may be fruitful for educators in PGME, is the new position paper of the Canadian Psychiatric Association’s (2023). This paper outlines practices in psychiatry at the micro (individual/clinical), meso (professional/institutional), and macro (social/ideological) levels, and calls for “training at all levels to facilitate working with racialized individuals, families, and communities through collaboration and collective empowerment” (p.6). Drawing on a framework that clearly delineates the three levels where anti-racist pedagogy may be implemented may be a valuable approach for advancing anti-racist education in PGME. However, while clearer conceptualizations are helpful, they do not automatically lead to changes in professional behaviour. Smith et al (2021) highlight many issues of structural racism and its impact on health, such as disparities in waiting times, access to care, lack of interpreter services, or technological bias (i.e., inaccuracy of pulse oximetry in darker skin tones). While exposure to examples of structural racism are essential for raising awareness, there continues to be a gap in how this knowledge translates into meaningful professional action.

### Design of education interventions

The findings suggest that many antiracist education interventions are in the early stages of development. The interventions vary in scope and scale, appear at many stages of the curriculum, use different methods of assessment and evaluation, and target theoretical knowledge and practical clinical skills in diverse ways. Of note, many are trainee-led, consistent with our observation that many faculty members have not been mentored in clinical antiracism education, and the work is largely exploratory.

This raises questions about how to integrate anti-racism educational interventions into the structure of PGME programs when expertise may not be available in the clinical setting. In particular, more research is needed on the design of educational interventions that move medical residents from knowledge of health inequities caused by racism to building skills to address them. Guidance from outside of the medical professions, such as racialized community and education scholars, may be instructive.

Adding anti-racist educational interventions to typically overloaded PGME curricula would require strategic, innovative pedagogical design and delivery. The review findings suggest that integrative curricular interventions had stronger content and pedagogical design elements and devoted more time to the application of the skills and tools taught. Thus, it seems important to consider ways of integrating anti-racism as a praxis as a practice across the curriculum, rather than as a ‘one-off’ intervention. The findings point to the importance of including time for residents to ‘practice’ the application of anti-racist behaviors and principles, beyond mere cognitive understanding.

The concept of ‘retention of learning’ is discussed in a scoping review of cultural competency curricula in US medical education (Atkinson et al., 2022), which found that many studies did not evaluate longitudinally over many years, and those that did showed that residents’ awareness of their own privilege declined significantly over time. This decline may also be influenced by institutional culture, behaviour, and commitment to anti-racist practice. The implications for anti-racist educational design suggest that it may be critical to maintain longitudinal curricula throughout the course of residency. Some ideas identified in the review were journal clubs where residents share and discuss anti-racist resources, or spaces to discuss varied perspectives on medical cases through an anti-racist framework. This educational challenge is ripe for further research.

Sustained mentorship is also key, such as investing institutional resources in faculty development (Garvey et al., 2022). Sustainable structural changes can include diversity committees, strengthening mentorship for fellows, and developing support structures for trainees who experience racism and discrimination (Karvonen et al. (2022). To enact a structural approach to change the diversity committee described by Karvonen et al. (2022) “organizes DEI-focused professional development workshops, provides diverse search committees for faculty positions, and explicitly values DEI work in faculty advancement” (p.363). There is value in an embedded, structural framing of educational interventions as a pedagogical approach, with a demonstrated commitment to address systemic or structural racism across the organization in meaningful ways, as discussed above.

### Goals of the interventions

Another key misalignment identified in the data was the distinction between imparting *knowledge* about the impacts of racism, and developing *practice-based skills* that address it. While residency requires a rigorous academic framework, it is also an apprenticeship in learning how to *practice* medicine, and moving from theoretical knowledge of medicine to its practical application is a fundamental basis of its pedagogy. Indeed, one of the key takeaways from Atkinson et al.,’s 2022 paper is the harm done by overloading trainees with information for which they have no tools.

Research that examines the translation of theoretical knowledge to practice is particularly important to show what competence in antiracist approaches might look like, and how practice-oriented pedagogies can be integrated into curricula (Osei-Tutu et al., 2023). Future work needs to address which skills are most critical to have a lasting impact on physician practice and a tangible effect on health outcomes or patient experience. While several interventions focus on developing interpersonal communication skills across many “cultures” to achieve a better therapeutic relationship, and thus presumably more effective diagnosis and negotiation of treatment plans (Kokas et al., 2019, Mendizabal et al., 2021, Diaz et al. 2016, Sherman et al., 2019), this limited view of the clinical encounter as simply an exchange of information fails to address other skills that are critical. For example, Garvey et al. for instance emphasize that critical appraisal of the literature is a skill that has a direct impact on the clinical encounter, by shifting the clinician’s understanding of what evidence applies to the patient’s situation. Emery et al. (2022) consider building skills in community organizing, although their focus on physician leadership may risk perpetuating pre-existing power dynamics. A more interesting focus of skills-building, for instance, may be on how to develop skills in community partnership in developing and delivering antiracist curricula.

### Who benefits?

Another question that remains is the role of the communities that use the health services that the trainees will provide. A key finding of this review is the almost complete absence of the communities meant to benefit from these changes from the design, implementation, or evaluation of these curricular changes, at least as reported in the studies. While there are some studies that demonstrate the benefits of active partnership with community groups and members in the design and delivery of medical education, (Omotara et al., 2006; Takamura et al., 2017), the literature tends to address community engagement primarily at other levels of health programming, such as recruitment of practitioners and implementation of public health initiatives, rather than educational benefits.

Given that the majority of these initiatives aim to address disparities affecting particular communities, there is clearly a need for more work to consider how community members can contribute to the design, delivery, and evaluation of anti-racism educational interventions.

In the current review, Kesler et al (2015) demonstrates the most developed interface with community organisations and teachers with lived experience, while several other studies mention the realities of the communities they serve as a foundation for their work (Smith et al., 2021, Diaz et al., 2016, Karvonen et al., 2022, Mendizabal et al., 2021, Kokas et al., 2019, Neff et al., 2017). Future anti-racism education work would do well to explicitly explore the priorities and perspectives of communities of the health services they work with, and to elicit their expectations of what trainees should learn to address the disparities they experience.

### Social accountability: Who should be evaluating the interventions?

Evaluation techniques are critical to becoming more effective in undoing the inequities communities experience. The findings point to a clear misalignment between the objectives of educational interventions and the predominant methods of evaluation. In 2010, Mian et al. wrote critically that “although the evaluation of a curriculum is typically regarded as important, there has been no consensus on how best to determine the impact of a cultural competency curriculum” (p.826). Thirteen years later, this appears to hold true for anti-racism curricula, whose standard evaluation lie predominantly within self-reported Likert scale surveys.

Fifteen out of 17 evaluations of residency educational interventions were completed by participants themselves. We question whether this dominant paradigm of self-reported outcomes provides a rigorous measure of the effectiveness of anti-racism education in medical education, especially when hampered by low response rates and small sample sizes, as in this review. There is also the issue of social accountability in this work: who should be evaluating the interventions? Who is this change in practice accountable to? Who does this practice impact? Should the communities impacted by healthcare inequities be the ones evaluating these interventions or should it be residents themselves? Self-evaluation is arguably an inadequate measure for advancing social accountability.

Promising were the few innovative outcome measures that focused on impact and action in the clinical context. For example, studies that used surveys and focus groups to collect feedback on post-training impact on clinical practice, and approaches that asked residents, “How will you incorporate what you learned during this monthly meeting into your project, studies, and other aspects of your life”. Future educational evaluations that focus on changes in actions and behaviours in the clinical setting, and the longevity of impact, are essential for advancing the field.

### The work of anti-racist education

It is crucial to recognize the demands and potential burdens that fall on those who do this work. Karvenon et al. (2022) raise integral points related to the emotional exhaustion of leading DEI and anti-racism work, especially when the residents leading this work are women of colour who may experience discrimination throughout such processes. Thus, the complexities of working in this space point to the potential need for emotional and institutional support, as well as further research, critical thinking, and active imagination about possibilities for the future.

Working toward holistic, sustainable, and structural change within organizations echoes the questions that renowned anti-racist education scholar George Dei (2014) poses to those doing anti-racist work: “Who is teaching in our universities, and how is our curriculum diversified to ensure that we are telling multiple stories? How are we making the knowledge and education relevant to the communities from where we draw our students?” (p.243). These kinds of questions are essential to guiding the design and research of anti-racist education of the future.

### Limitations

This scoping review has a few limitations. Our search strategy was limited to articles published in English, so we may have overlooked many non-English articles. We also restricted the publication date to after 2010; however, there may have been many articles published well before then. We also recognize that many interventions have been developed, but not yet documented, so this may not be an accurate representation of what is currently instituted at medical faculties. Lastly, our definition of systemic and structural racism was implicitly shared among our team and perhaps taken for granted, as we assumed that certain keywords and phrases alluded to this language. If we had screened for articles that explicitly named these terms, we would have ended up with a much smaller pool of articles. At the same time, some interventions may have included some teachings about systemic racism, but conceptualized it differently in their descriptions, leading it to be excluded during screening.

## Conclusion

While there are limitations, we appreciate the studies in this scoping review for leading the way in anti-racist work through educational interventions within their respective capacities. Collectively, these studies advance our knowledge of how anti-racist educational interventions are conceptualized, designed, implemented, and evaluated in post-graduate medical education, and provide important insights for future education, practice, and research. It is our hope that educators and scholars will continue to engage in dialogue across the field, seeking greater alignment between our stated educational goals and our educational practices, as well as more holistic, sustainable, and structural approaches to anti-racist education in the future that respond to community needs.

## Supplementary Information

Below is the link to the electronic supplementary material.

Supplementary file1 (DOCX 35 KB)
