# Supplementary material for: Towards anti-racist futures: a scoping review exploring educational interventions that address systemic racism in post graduate medical education
Source: Adv Health Sci Educ Theory Pract. 2024 Jun 14;30(2):359–81. doi: 10.1007/s10459-024-10343-1 (PMC11965230; doi:10.1007/s10459-024-10343-1)
Supplement: Supplementary file 1 — Supplementary file1 (DOCX 35 KB) [file 10459_2024_10343_MOESM1_ESM.docx]

| **Full Citation, Country of Study**  (paper#), and  **Title** | **Aim of Study** | **Postgraduate Medical Specialty** | **Study Design & Evaluation Method** | **Description of Educational Intervention** |
| --- | --- | --- | --- | --- |
| Drum, B. M., Sheffield, C. R., Mulcaire-Jones, J., Gradick, C., & Mulcaire-Jones, J. P. (2021). Formation and evaluation of an academic elective for residents in a combined internal medicine-pediatrics residency program. Cureus, 13(7).  USA, [#1](https://drive.google.com/file/d/1vU-GxDK0nWFK8OwUh93CaTBOEj066ykH/view?usp=share_link) | Create, implement, and evaluate an academic elective with dedicated time for upper-level residents to develop and utilize valuable skills in teaching, evidence appraisal, wellness, and anti-racism. | Combined Internal Medicine & Pediatrics | Intervention development and implementation, with post-intervention survey evaluation | - Four-week academic elective to learn and explore the four domains of resident teaching, evidence-based clinical practice, wellness, and anti-racism work. - Included several clinical sessions dedicated to implementing the skills taught in the elective. |
| Smith, K. J., Harris, E. M., Albazzaz, S., & Carter, M. A. (2021). Development of a health equity journal club to address health care disparities and improve cultural competence among emergency medicine practitioners. AEM Education and Training, 5, S57-S64.  USA, [#2](https://drive.google.com/file/d/1juSqEArJNL8kmx3WowHPC6kULI-D5QW8/view?usp=share_link) | Describe the development and implementation of a Health Equity Journal series. | Emergency Medicine | Intervention development and implementation, no evaluation | - Monthly SDOH- and cultural competency-based health equity journal club, with content mapped to the ACGME program, core competency milestone requirements, and CLER mandates. - Four educational domains developed: racial/ethnic disparities in health care, gender disparities in health care, LGBTQ+ health care disparities, SDOH. |
| Díaz, E., Armah, T., Linse, C. T., Fiskin, A., Jordan, A., & Hafler, J. (2016). Novel brief cultural psychiatry training for residents. Academic Psychiatry, 40, 366-368.  USA, [#3](https://drive.google.com/file/d/1n0OHM03BpCx0I1vguoqxgm4FZWsgszhF/view?usp=share_link) | Describes the design and implementation of an innovative cultural psychiatry curriculum. | Psychiatry | Intervention development and implementation, no evaluation | - Four 90-minute training sessions, including a list of reading materials - Session 1 - implicit assumptions influencing clinical interactions; Session 2 - how bias and prejudices impact health disparities; Session 3 - practice strategies that aim to elicit cultural information; Session 4 - Develop self-awareness and feedback skills. |
| Karvonen, K. L., Menjívar-López, J. S., Brissett, D., McBride, D., Olveda, R., Fabersunne, C. C., ... & Argueza, B. R. (2022). A resident-led initiative to advance diversity, equity, inclusion, and antiracism in a pediatrics residency program. Academic Pediatrics, 22(3), 360-364.  USA, [#4](https://drive.google.com/file/d/1_w_qGUOjJOIDC85b9nfZmGgsTBPuBrNE/view?usp=share_link) | Discuss the implementation and key outcomes of curriculum initiatives over the past 6 years, as well as challenges and lessons learned, from the unique perspective of a group led primarily by residents who are women of color. | Pediatrics | Intervention development and implementation | - Longitudinal curriculum on DEI and antiracism, including didactics (about cultural humility, structural racism, stereotype threat, leading a diverse team), standardized simulations on interrupting microaggressions, and racial affinity groups. |
| Johnson, C., Rastetter, M., & Olayiwola, J. N. (2022). Pathways to equity: A pilot study implementing a health equity leadership curriculum in residency education as an antidote to systemic racism. Journal of the National Medical Association, 114(2), 141-146.  USA, [#5](https://www.sciencedirect.com/science/article/abs/pii/S0027968421002388?via%3Dihub) | Assess the impact of a resident-led health equity curriculum on the self-efficacy of family medicine residents in caring for vulnerable populations and managing challenging patient scenarios. | Family Medicine | Intervention development and implementation, with pre- and 6-month post- implementation survey evaluation | - Biweekly resident-led sessions for 18 months designed to deepen residents’ knowledge and understanding of health disparities, social inequities, and community involvement, utilizing validated resources from the EveryONE Project Health Equity Toolkit created by the American Academy of Family Physicians’ Center for Diversity and Health Equity. - 4 main themes: SDOH, cultural humility, narrative medicine, physician advocacy. |
| Simpson, T., Evans, J., Goepfert, A., & Elopre, L. (2022). Implementing a graduate medical education anti-racism workshop at an academic university in the Southern USA. Medical Education Online, 27(1), 1981803.  USA, [#6](https://drive.google.com/file/d/14p_1dDGETI2JSFbF8WqMScCQ2MnWo7UA/view?usp=share_link) | Describe the development of an anti- racism workshop to raise awareness on the  impact of racism in healthcare and provide tools that  individuals can utilize to dismantle racism. | Postgraduate Medical Education – no particular specialty | Intervention development and implementation, with postworkshop survey evaluation | - A 1.5-hour interactive workshop covering content about microaggressions, colorblindness, tokenism, stereotypes, levels of racism, the impact of racism on health, and anti-racism concepts. |
| Jindal, M., Mistry, K. B., McRae, A., Unaka, N., Johnson, T., & Thornton, R. L. (2022). “It Makes Me a Better Person and Doctor”: A Qualitative Study of Residents’ Perceptions of a Curriculum Addressing Racism. Academic pediatrics, 22(2), 332-341.  USA, [#7](https://drive.google.com/file/d/1Mdsm5w0ptCuXprUCU55ewUdkUaDdbDbv/view?usp=share_link) | Explore how pediatric residents perceive the  impact of a curriculum addressing racism on their knowledge,  motivation, skills and behaviors, and investigate the contextual  factors that promote or impede the curriculum’s effectiveness. | Pediatrics | Intervention development and implementation, with semi-structured interview evaluation | - Three 1-hour lectures with reflective discussion, each titled “Self-Reflection on Implicit Bias”, “Historical Trauma”, and “Structural Racism”. - Prior to curriculum, residents take the IAT to assess their unconscious attitudes. |
| Willen, S. S., Bullon, A., & Good, M. J. D. (2010). Opening up a huge can of worms: reflections on a “cultural sensitivity” course for psychiatry residents. Harvard review of psychiatry, 18(4), 247-253.  USA, [#8](https://drive.google.com/file/d/1kk1cAL78z7qMIkAAGr6uOvGePjBENL5Z/view?usp=share_link) | Explore some challenges and dilemmas that cultural sensitivity courses can easily generate. | Psychiatry | Intervention description and implementation, with pre- and post-course semi-structured interviews & course observation evaluation | - Eighteen 1-hour weekly sessions aimed at increasing resident’s awareness of the cultural, social and economic factors that influence the diagnosis and treatment of mental health disorders in order to have a clinical intervention that is congruent, fair and effective to the patient’s reality. |
| Garvey, A., Lynch, G., Mansour, M., Coyle, A., Gard, S., & Truglio, J. (2022). From race to racism: teaching a tool to critically appraise the use of race in medical research. MedEdPORTAL, 18, 11210.  USA, [#9](https://drive.google.com/file/d/1CnQEEwazVtb_hVDoU2sMqGu4fwtzpVR1/view?usp=share_link) | Showcase development of the Critical Appraisal of Race in Medical Literature (CARMeL) tool to help learners assess the use of race in medical research. | Internal Medicine | Intervention description and implementation, with pre- and post-workshop survey evaluation | - One-time workshop which started with a discussion of race as a sociopolitical construct and methodological pitfalls of the use of race in medical research. - Facilitators presented the CARMel tool then presented the CARMeL tool, which considers the use of race from the lens of internal validity and external validity, with the final step centering on applicability to patients and the societal implications of the application of these results. |
| Emery, E. H., Shaffer, J. D., McCormick, D., Zeidman, J., Geffen, S. R., Stojicic, P., ... & Basu, G. (2022). Preparing doctors in training for health activist roles: a cross-institutional community organizing workshop for incoming medical residents. MedEdPORTAL, 18, 11208.  USA, [#10](https://drive.google.com/file/d/1EEH0QMprSdSwYxhZeiWx1zfwNBYDpI0_/view?usp=share_link) | To describe the development of a cross-institutional workshop and evaluating whether training in community organizing would be a feasible and effective method to introduce residents to key leadership skills. | Postgraduate Medical Education – no particular specialty | Intervention description and implementation, with post-workshop survey evaluation | - 3-hour, cross-institutional workshop prior to intern orientation, introducing community organizing as a framework for effective physician advocacy - Utilized didactic sessions, video examples, and small-group practice led by trained coaches to familiarize participants with one community organizing leadership skill—public narrative—as a means of creating the relationships that underlie collective action. |
| Martinez, S., Araj, J., Reid, S., Rodriguez, J., Nguyen, M., Pinto, D. B., ... & Mason, H. (2021). Allyship in residency: an introductory module on medical allyship for graduate medical trainees. MedEdPORTAL, 17, 11200.  USA, [#11](https://drive.google.com/file/d/1Up5Yxs1zuY1gU4s9Epig-qbWgj1ftGBi/view?usp=share_link) | To propose use of allyship to increase graduate medical trainee understanding of diversity and focus on health equity. | Pediatrics, Family Medicine, Emergency Medicine, and Surgery | Intervention description and implementation, with pre- and post-workshop survey evaluation | - 1-hour workshop aimed at helping residents understand the definition of allyship, effective allyship to patients and colleagues, and allyship differences across communities. - Utilized a didactic presentation module and facilitated case study discussions |
| Mian, A. I., Al-Mateen, C. S., & Cerda, G. (2010). Training child and adolescent psychiatrists to be culturally competent. *Child and Adolescent Psychiatric Clinics*, *19*(4), 815-831.  USA, [#12](https://drive.google.com/file/d/1F4DkSEoqX4vnQJtvcME32P48WiqIeJLR/view?usp=share_link) | To explain the role of culture and cultural competence in  clinical care, and outline components of a cultural sensitivity curriculum. | Psychiatry | Theoretical Article | N/A |
| Chary, A. N., Molina, M. F., Dadabhoy, F. Z., & Manchanda, E. C. (2021). Addressing racism in medicine through a resident-led health equity retreat. Western Journal of Emergency Medicine, 22(1), 41.  USA, [#13](https://drive.google.com/file/d/17YTHmsocwXKud_bbD-08oaRRTZZgedSP/view?usp=share_link) | To describe an innovative health equity retreat to teach about forms of racism and skills for responding to racial inequities in clinical environments, | Emergency Medicine | Intervention description and implementation, with post-retreat survey evaluation | - A 3-hour retreat to raise awareness of race-based inequities in patient and resident experience, and to build skills in recognizing and addressing racial inequities and microaggression - Retreat aimed to encourage peer discussions about racism and the potential roles of clinicians, bystanders, and allies in promoting equitable patient care |
| Mendizabal, A., Fan, J. H., Price, R. S., & Hamilton, R. H. (2021). Feasibility and effectiveness appraisal of a neurology residency health equities curriculum. *Journal of the Neurological Sciences*, *431*, 120040.  USA, [#14](https://drive.google.com/file/d/1ekwNQYKf-s-38ZuVVwMWjRuyM2dTH1E6/view?usp=share_link) | To demonstrate the implementation of a health equities curriculum for neurology residents | Neurology | Intervention description and implementation, with pre- and post-curriculum survey evaluation | - A 7 lecture curriculum implemented over one year, to explore healthcare delivery within the context of social determinants of health in urban communities. - Topics included: SDOH, healthcare disparities, cultural humility, implicit bias, and addressing disparities as independent practitioners. |
| Hammond, B. A., & James, C. (2020). Creating an intergroup dialogue curriculum on race for psychiatry residents. *Academic Psychiatry*, *44*(4), 498-499.  USA, [#15](https://drive.google.com/file/d/1GoYH0M39jK14l6kmWn6w7MrkbRnrHmvV/view?usp=share_link) | To explain the creation of an intergroup dialogue curriculum on race for psychiatry residents | Psychiatry | Letter to the editor – Intervention description | - Six 90-minute sessions consisting of intergroup dialogue where participants prepare testimonials that describe their experience with race in their lives. |
| Kokas, M., Fakhoury, J. W., Hoffert, M., Whitehouse, S., Van Harn, M., & Baker-Genaw, K. (2019). Health care disparities: a practical approach to teach residents about self-bias and patient communication. *Journal of Racial and Ethnic Health Disparities*, *6*, 1030-1034.  USA, [#16](https://drive.google.com/file/d/13eJPnfk_BFb_GIr-a6z6vWqq5LGpeYcq/view?usp=share_link) | To demonstrate the health care disparities (HCDs) program | Postgraduate Medical Education – no particular specialty | Intervention description and implementation, with 4 assessments of modified RACE survey evaluation | - 2 hr program, whose focus was on understanding of one’s self-bias and how to use open-ended questions to uncover the patient’s personal challenges and constraints. |
| Dennis, S., Gold, R., & Wen, F. (2019). Learner reactions to activities exploring racism as a social determinant of health. *Family medicine*, *51*(1), 41-47.  USA, [#17](https://drive.google.com/file/d/13MTue4ShprISvc2YGG3InQ7DrnIzmea-/view?usp=share_link) | To describe the responses of learners to learning sessions on racism  as a social determinant of health (SDOH) highlighting structural, personallymediated, and internalized racism. | Family and Community Medicine | Intervention description and implementation, with quantitative and qualitative surveys (written comments) evaluation | - Weekly sessions to enhance learners’ understanding that individual experiences are embedded within a social-cultural context through lectures, workshops, and guided museum tours. - Topics covered include three levels of racism, race and equity, privilege and intersectionality, and implicit bias. |
| Sherman, M., Ricco, J., Nelson, S., Nezhad, S., & Prasad, S. (2019). Implicit bias training in a residency program: aiming for enduring effects. *Family medicine*, *51*(8), 677-681.  USA, [#18](https://drive.google.com/file/d/10QlokDba9-TzYXFecyPagmMXn2pCtnrE/view?usp=share_link) | To offer and evaluate parallel trainings  for residents and faculty by a national expert. | Family Medicine | Intervention description and implementation, with external evaluator-led focus group evaluation | - Two 60-90 minute parallel workshops focused on both patient care and teaching, and how to incorporate practical, applied recommendations to address implicit bias in practice. |
| Zeidan, A., Tiballi, A., Woodward, M., & Di Bartolo, I. M. (2020). Targeting implicit bias in medicine: lessons from art and archaeology. *Western Journal of Emergency Medicine*, *21*(1), 1.  USA, [#19](https://drive.google.com/file/d/19Iy7TPkkEGOl1Poq3TMl7YZKGrTrvhSl/view?usp=share_link) | Provide a description of an educational approach using archaeologic concepts to introduce implicit bias to trainees. | Internal Medicine & Emergency Medicine | Intervention description and implementation, with pre- and post-survey evaluation | - Innovative exercise introducing implicit bias in a non-traditional environment with 3 components: a pre-brief with session leaders, viewing three objects using a tool called "deep description”, and a post-session reflective discussion. |
| Neff, J., Knight, K. R., Satterwhite, S., Nelson, N., Matthews, J., & Holmes, S. M. (2017). Teaching structure: a qualitative evaluation of a structural competency training for resident physicians. *Journal of General Internal Medicine*, *32*, 430-433  USA, [#20](https://drive.google.com/file/d/1wRRhhjXJUipyzg5NvYFLgswrO0GCv-2i/view?usp=share_link) | Describes the development, implementation, and evaluation of a structural competency training for medical residents. | Family Medicine | Intervention description and implementation, with post-training qualitative survey and focus group | - A 3 hour session for residents to recognize and develop skills to respond to illness and health as the downstream effects of social, political, and economic structures. - Topics include: how structures affect patient health, how structures affect the clinical encounter, and brainstorming strategies to use beyond the clinic. |
| Paroz, S., Daele, A., Viret, F., Vadot, S., Bonvin, R., & Bodenmann, P. (2016). Cultural competence and simulated patients. *The clinical teacher*, *13*(5), 369-373.  Switzerland, [#21](https://drive.google.com/file/d/1pJbYTzCJ0XOVXFe4OBm3mmYgRx5Uo2Ws/view?usp=share_link) | To describe and test the feasibility of cultural competence  training with simulated patients. | Postgraduate Medical Education – no particular specialty | Intervention description and implementation, with post-training survey and education expert-led focus group evaluation | - Two 45-minute complementary case scenarios based on real clinical practice and focused on specific clinical skills, carried about two simulated patients, a group debriefing, and a post-debriefing role ‘replay’. |
| Kesler, D. O., Hopkins, L. O., Torres, E., & Prasad, A. (2015). Assimilating traditional healing into preventive medicine residency curriculum. *American Journal of Preventive Medicine*, *49*(5), S263-S269.  USA, [#22](https://drive.google.com/file/d/173RWQ0uYo2u0HVTzeJmmIiP_oSbXjrrP/view?usp=share_link) | To describe the culturally based traditional healing curriculum that has been implemented in the University of New Mexico Public Health and General Preventive Medicine Residency Program in order to fulfill this knowledge necessity. | Public Health and General Preventive Medicine | Intervention description and implementation, with resident performance (evaluation, examination), program quality (resident evaluation of activities), and graduate performance (quality improvement survey) evaluation | 1. An interactive course at the UNM main academic  campus titled “Traditional Medicine Without Borders:  Curanderismo in the Southwest and Mexico”;  2. Progressive practicum rotations, “Introduction to Health Disparities in NM and Beyond” to introduce the resident to community health organizations that incorporate cultural learning into their services followed by a 4-week practicum rotation, “Emerging to Health Equity,” which allowed residents to be the educators;  3. A practicum rotation at the UNM Center for Life (CFL), a clinically based integrative medicine center; and  4. Applicable online curriculum from the Arizona Center for Integrative Medicine (AzCIM). |
| Atkinson, R. B., Khubchandani, J. A., Chun, M. B., Reidy, E., Ortega, G., Bain, P. A., ... & Smink, D. S. (2022). Cultural competency curricula in US graduate medical education: a scoping review. *Journal of Graduate Medical Education*, *14*(1), 37-52.  USA, [#23](https://drive.google.com/file/d/15q3dDzcuO5vH68CUB-xEMvHX1DsoO9-V/view?usp=share_link) | To determine what cultural competency curricula exist specifically in GME. | Postgraduate Medical Education – no particular specialty | Scoping Review | N/A |
